# Remission Is Maintained after Switch from Dose-Optimised Intravenous Treatment to Subcutaneous Treatment with Vedolizumab in Inflammatory Bowel Disease

**DOI:** 10.3390/medicina60020296

**Published:** 2024-02-09

**Authors:** Špela Pintar, Jurij Hanžel, David Drobne, Matic Koželj, Tina Kurent, Nataša Smrekar, Gregor Novak

**Affiliations:** 1 Department of Gastroenterology, University Medical Centre Ljubljana, Japljeva 2, 1000 Ljubljana, Slovenia; spela.pintar@kclj.si (Š.P.); jurij.hanzel@kclj.si (J.H.); david.drobne@kclj.si (D.D.); matic.kozelj@kclj.si (M.K.); tina.kurent@kclj.si (T.K.); natasa.smrekar@kclj.si (N.S.); 2 Faculty of Medicine, University of Ljubljana, Vrazov trg 2, 1000 Ljubljana, Slovenia

**Keywords:** inflammatory bowel disease, Crohn’s disease, ulcerative colitis, vedolizumab, subcutaneous formulation

## Abstract

*Background and Objectives*: The subcutaneous (SC) formulation of vedolizumab has proven to be effective for the maintenance of remission after intravenous induction. Little is known about the efficacy of switching from intravenous maintenance treatment to SC. We aimed to assess the real-world efficacy of switching to SC treatment and to assess the impact of a baseline treatment regimen. *Materials and Methods*: In this observational cohort study, adult patients with inflammatory bowel disease who were switched to SC vedolizumab maintenance treatment were enrolled. Patients after intravenous induction and patients who switched from intravenous maintenance treatment (every 8 weeks or every 4 weeks) were included. The SC vedolizumab dosing was 108 mg every 2 weeks, regardless of the previous regimen. The clinical, biochemical, and endoscopic disease activity parameters and vedolizumab serum concentrations at the time of the switch and at the follow-up were assessed. *Results*: In total, 135 patients (38% Crohn’s disease, 62% ulcerative colitis) were switched to SC vedolizumab treatment. The median time to the first follow-up (FU) was 14.5 weeks (IQR 12–26), and the median time to the second FU was 40 weeks (IQR 36–52). Nine patients (7%) discontinued SC vedolizumab treatment, with two-thirds of them discontinuing due to active disease. In all dosing regimens, there were no significant changes in the clinical scores and CRP at the baseline and first and second FUs. Clinical and biochemical remission appeared to be maintained irrespective of the previous dosing regimen. *Conclusions*: The results of this real-world study suggest that the maintenance of clinical and biomarker remission can be achieved in patients who switched from intravenous to SC vedolizumab. The baseline vedolizumab dosing regimen (every 4 weeks versus every 8 weeks) did not have an impact on outcomes.

## 1. Introduction

Inflammatory bowel disease (IBD) is a chronic inflammatory disorder of the gastrointestinal tract that most commonly manifests with bloody diarrhoea and abdominal pain. If the inflammation is uncontrolled, it can cause progressive functional and structural damage and impair patients’ quality of life. Among the current therapeutic armamentarium, several small molecules and biological agents are available, including vedolizumab [1].

Vedolizumab is a monoclonal antibody that targets α4β7 integrin, which is preferentially expressed on gut-homing lymphatic cells and prevents their trafficking into the inflamed gut. The gut-selective mechanism of action contributes to vedolizumab’s favourable benefit–risk profile [2,3].

Vedolizumab has been registered as an intravenous (IV) induction and maintenance treatment for ulcerative colitis (UC) [4] and Crohn’s disease (CD) [5]. The recommended dose regimen of vedolizumab is 300 mg administered via intravenous (IV) infusion at weeks 0, 2, and 6, followed by infusions every 8 weeks thereafter. In the case of a loss of response or an inadequate response, dose escalation by shortening the dosing interval to every 4 weeks was proven effective in approximately half of the patients [6,7]. Recently, a subcutaneous (SC) formulation of vedolizumab has been approved for maintenance treatment after showing efficacy and safety in the phase III clinical trials VISIBLE 1 [8] and VISIBLE 2 [9]. The serum levels of vedolizumab were higher with SC administration compared to IV. Both clinical trials evaluated SC maintenance treatment (108 mg every 2 weeks) in patients who responded at week 6 to an induction with two infusions of vedolizumab [8,9]. However, these clinical trials did not assess IBD patients who were treated with maintenance IV vedolizumab before transitioning to SC.

Switching from IV infusion to SC injections is an appealing option due to the potential for self-administration at home, which could enhance patient convenience [10]. Additional advantages include a shorter application time, a decreased incidence of infusion-related adverse events, an improved quality of life, a reduction in the time needed to travel to a healthcare institution, and a decrease in healthcare system costs [10,11]. Conversely, more frequent injection-site reactions are observed with SC applications compared to IV applications [8]. Nevertheless, most patients and healthcare professionals express a preference for SC application over IV [10,11]. There are limited data about switching from IV maintenance to SC treatment. Four prospective real-world studies from Europe reported that transition to SC maintenance treatment is feasible, effective, and safe [12,13,14,15]. Data regarding switching from IV maintenance to SC treatment in patients who were previously dose-optimised due to a loss of response or an inadequate response by shortening the IV dosing interval to every 4 weeks are even more deficient. 

In our study, we aimed to assess the drug persistence, efficacy, and pharmacokinetics of switching to an SC vedolizumab formulation in a real-world cohort of IBD patients and to assess if the baseline IV regimen (maintenance every 8 weeks (q8), maintenance every 4 weeks (q4), or IV induction) impacts the outcomes after the transition.

## 2. Materials and Methods

### 2.1. Study Design and Population

This observational study was conducted in a tertiary referral IBD centre (University Medical Centre Ljubljana, Slovenia) following the rules of the Declaration of Helsinki of 1975, revised in 2013. Ethical permission was granted by the Slovenian National Ethics Committee (0120-013/2016-2). Consecutive prospectively followed adult patients with IBD who were switched to SC vedolizumab maintenance treatment from May 2021 onward were enrolled. Follow-up lasted until July 2022. Therefore, the duration of follow-up differs among enrolled patients. All patients being treated with vedolizumab (with response after IV induction or undergoing IV maintenance treatment) were offered the option of switching to SC formulations after an exact explanation of known data. 

The included patients had to be ≥18 years of age with a confirmed diagnosis of IBD (UC, CD, or unclassified IBD) and undergoing treatment with SC vedolizumab—either after IV induction or after IV maintenance. There were no exclusion criteria. Demographics and baseline characteristics were extracted from medical files, including age, gender, diagnosis, disease duration, disease phenotype, smoking status, weight, height, previous biological therapy, previous exposure to corticosteroids, and duration of IV vedolizumab therapy before switching to SC. 

The included patients were grouped based on the IV treatment regimen before switching to SC: IV maintenance treatment every 8 weeks (q8 cohort), optimised IV maintenance treatment every 4 or 6 weeks (q4 cohort), and IV induction (two or three IV infusions). After the switch to SC, all groups were treated with the same SC vedolizumab regimen (108 mg every 2 weeks), regardless of the previous IV regimen. Follow-up visits were individually scheduled by the treating physician; therefore, not all patients had visits at the same time point. Data on SC vedolizumab discontinuation, clinical and endoscopic scores, and biochemical parameters (including vedolizumab serum concentrations) were documented at baseline and throughout follow-up.

### 2.2. Study Endpoints and Definitions

The main outcome was the proportion of patients who discontinued SC vedolizumab. Drug discontinuation could be due to disease activity, intolerance or side effects, the need for IBD surgery or hospitalisation, patients’ wishes, moving to another IBD centre, and being lost to follow-up. The discontinuation date was defined as the day of the last SC vedolizumab application.

Additional outcomes included clinical remission, biochemical remission, and endoscopic remission after switching to SC treatment. Vedolizumab serum concentrations were assessed at the time of switch and at follow-up visits. 

Clinical disease activity was assessed using the Harvey Bradshaw index [16] (HBI) for CD or the partial Mayo score [17] (pMayo) for UC. Clinical remission was defined as HBI < 5 in CD and pMayo < 2 in UC.

Fecal calprotectin (FC), C-reactive protein (CRP), and vedolizumab serum concentration were measured at the time of switch and at follow-up visits. FC was measured using the Calprest assay (Eurospital, Trieste, Italy) and CRP was measured with the ADVIA 1800 Chemistry System (Siemens, Germany). Biochemical remission was defined as CRP < 5 mg/L and FC < 100 µg/g [18]. Vedolizumab concentrations were measured with the Conformité Européenne-marked apDia vedolizumab enzyme-linked immunosorbent assay (Turnhout, Belgium) with a measurement range between 1 and 50 µg/mL.

The endoscopies were scheduled by the treating physician. Disease activity was assessed using the endoscopic Mayo score [19] in UC and by the presence of ulcers in CD. Endoscopic remission was defined as a Mayo endoscopic score < 2 in UC and an absence of ulcers > 5 mm in CD. 

### 2.3. Statistical Methods

All analyses were performed on a per-protocol basis. The continuous variables are presented as medians with interquartile ranges (IQRs). The vedolizumab serum concentrations before and after switching were compared using a paired Wilcoxon rank test. Values of *p* < 0.05 were considered statistically significant. All analyses were performed using IBM SPSS statistics for Windows, Version 21.0 (IBM Corp., Armonk, NY, USA).

## 3. Results

### 3.1. Patient Characteristics

In total, 135 patients were enrolled, 51 (37.8%) had CD, and 84 (62.2%) had UC (Table 1). The median time to the first follow-up visit was 14.5 weeks (IQR 12–26 weeks), and the median time to last follow-up was 40 weeks (IQR 36–52 weeks). No patients underwent dose escalation during the follow-up period. 

### 3.2. Drug Survival

A total of 9/135 patients (6.7%) discontinued SC vedolizumab treatment until the end of follow-up: 6 (7.1%) had UC and 3 (5.9%) had CD. Reasons for discontinuation were active disease with the need for treatment escalation in six patients, dysplasia at surveillance colonoscopy requiring colectomy in one patient, discontinuation due to the patient’s wishes in one patient, and loss to follow-up for one patient. The median time to treatment discontinuation was 22 weeks (IQR 14–45 weeks).

Out of the six patients who discontinued SC vedolizumab due to active disease, three were in the IV induction group and three were in the q4 group (Table 2). All were switched to another treatment. 

### 3.3. Clinical and Biochemical Disease Activity after Switch

The proportion of patients with CD in clinical remission was 17/18 (94%), 20/20 (100%), 19/11 (91%) in the q8 group, 7/11 (64%), 13/15 (87%), 5/7 (72%) in the q4 group, and 7/7 (100%), 7/7 (100%), 2/2 (100%) in the IV induction group at baseline and first and second follow-up, respectively. The proportion of patients with UC in clinical remission was 26/27 (96%), 25/28 (89%), 12/13 (92%) in the q8 group, 12/17 (71%), 15/17 (88%), 7/10 (70%) in the q4 group, and 13/18 (72%), 16/18 (89%), 9/9 (100%) in the IV induction group at baseline and first and second follow-up, respectively. In all groups of patients, there were no significant changes in clinical disease activity scores at baseline and first and second follow-up. Data for clinical disease activity are shown in Table 3.

Similar trends were noted with biochemical markers of disease activity (Table 3). In all groups of patients, there were no significant changes in CRP at baseline and first and second follow-up. Due to missing data, calculations were not performed for FC. 

**Figure 1:**
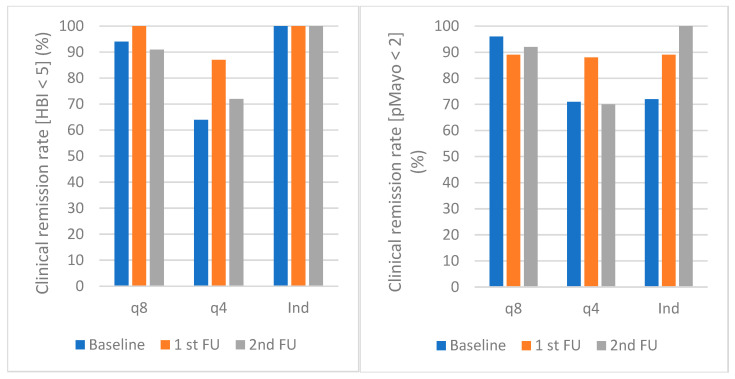
Clinical remission rates in CD (**left**) and UC (**right**) at baseline and first and second follow-up. Clinical remission was defined as HBI < 5 in CD and pMayo < 2 in UC. The median time to the first follow-up visit was 14.5 weeks (IQR 12–26 weeks), the median time to last follow-up was 40 weeks (IQR 36–52 weeks). Abbreviations: UC: ulcerative colitis; CD: Crohn’s disease; q8: intravenous vedolizumab every 8 weeks; q4: intravenous vedolizumab every 4 weeks; Ind: intravenous vedolizumab induction—2 or 3 infusions; HBI: Harvey Bradshaw index; pMayo: partial Mayo score.

**Figure 2:**
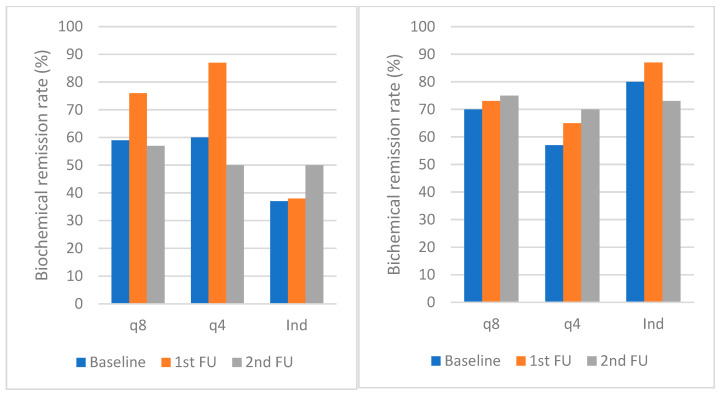
Biochemical remission rates in CD (**left**) and UC (**right**) at baseline and first and second FU. Biochemical remission was defined as CRP < 5 mg/L. The median time to the first FU visit was 14.5 weeks (IQR 12–26 weeks), the median time to last FU was 40 weeks (IQR 36–52 weeks). Abbreviations: UC: ulcerative colitis; CD: Crohn’s disease; q8: intravenous vedolizumab every 8 weeks; q4: intravenous vedolizumab every 4 weeks; Ind: intravenous vedolizumab induction—2 or 3 infusions; CRP: C-reactive protein; FU: follow-up.

Clinical proportions in biochemical remission rates in patients with CD and UC are presented in Figure 1 and Figure 2. 

### 3.4. Endoscopic Disease Activity after Switch

Endoscopic disease activity after the switch to SC formulation was assessed after a median time of 24.5 weeks (IQR 17–42), 29 weeks (IQR 21–42), and 21 weeks (IQR 16–29) in the q8, q4, and IV induction groups, respectively. Endoscopic remission in UC was reached in 89%, 67%, and 75% of patients in the q8, q4, and IV induction groups, respectively. 

The percentage of patients in endoscopic remission after the switch to SC formulation is presented in Table 4. 

### 3.5. Pharmacokinetics

The median vedolizumab serum concentration in each group at baseline and first and second follow-up is presented in Table 5. Vedolizumab serum concentration at first follow-up was significantly higher than at baseline in the q8 group (*p* < 0.001). However, no significant change in vedolizumab serum concentrations between baseline and first follow-up was observed in the q4 and IV induction groups. 

## 4. Discussion

To the best of our knowledge, this is the largest reported cohort of patients who transitioned from escalated 4-week IV vedolizumab dosing to SC vedolizumab. Our findings confirm the feasibility of switching to regular SC maintenance treatment (108 mg every 2 weeks) even in patients with more refractory disease, receiving the optimised dosing of IV vedolizumab. Notably, clinical and biochemical remission appear to be maintained after switching to the SC formulation, regardless of the previous IV treatment regimen.

Over a median follow-up of 40 weeks, only 9 out of 135 (7%) patients discontinued SC treatment with vedolizumab, with a median time to treatment discontinuation of 22 weeks. This contrasts with discontinuation rates of 27% and 39% within 52 weeks of treatment reported in the registration trials VISIBLE 1 and VISIBLE 2 [8,9]. However, the results of our study cannot be compared to randomised controlled trials due to different patient populations, variations in IV treatment regimens (notably, only induction with two infusions in VISIBLE trials) and variable follow-up time. A more reliable comparison can be made with other real-world studies on transitioning to SC treatment. In a study from Netherlands, 11.9% of patients discontinued treatment after a median follow-up of 27 weeks [12]. An English cohort reported an 8% discontinuation rate at week 12 [14]. Similarly, in a Swedish cohort, discontinuation rates at 6 and 12 months were 4.5% and 11.5%, respectively [15]. A Norwegian study reported a 7.4% discontinuation rate by week 26 [13]. These findings indicate comparable discontinuation rates to our results.

On the other hand, our findings indicate lower discontinuation rates compared to other real-world cohorts receiving IV vedolizumab maintenance treatment. For instance, in a French cohort, 7.5% discontinued treatment with vedolizumab by week 14 and 43.5% by week 54 [20]. In the long-term follow-up of the same study, the 1-, 2- and 3-year persistence rates of vedolizumab in patients with CD were 48.5%, 31.4% and 26.3% and in patients with UC, 61.0%, 49.9% and 42.9%, respectively [21]. In a Danish cohort, the 12-week, 52-week, and 17-month drug continuation rates were 81%, 61%, and 58%, respectively [22]. Finally, in the Dutch cohort, the probability of continuing receiving vedolizumab treatment after 52 and 104 weeks was 54.0% and 38.4% for CD and 60.8% and 51.3% for UC, respectively [23]. From these data, it appears that transitioning to the SC formulation might be both feasible and, at the very least, similarly effective in preventing treatment discontinuation. However, low discontinuation rates in our study might be explained by the long median duration of IV vedolizumab treatment in the q4 and q8 groups. It is possible that many non-responsive patients were already discontinued before transitioning to SC. 

The primary reason for treatment discontinuation in our study was active disease with the need for treatment escalation in two-thirds of patients. Interestingly, 4/6 of these patients were in clinical remission but had endoscopically active disease. In many of them, endoscopy was scheduled while still on IV maintenance treatment, meaning that disease could have been endoscopically active even before the switch to SC. Similar trends were observed in the Swedish cohort, where the majority discontinued due to active disease (5 out of 9) [15]. Conversely, in the Dutch cohort, most patients discontinued treatment due to adverse events (9/16) and a fear of needles (3/16). Only four (25%) discontinued treatment due to a loss of response [12]. Similarly, in the English cohort, 8/10 (80%) patients discontinued treatment due to adverse events [14]. Interestingly, none of the patients in our study discontinued treatment due to adverse events or a fear of needles. This could be attributed to the shared decision-making process allowing patients to choose between continuing IV treatment or transitioning to SC treatment. Moreover, all patients underwent comprehensive injection training by a specialised IBD nurse. 

The proportion of patients with UC and CD in clinical remission across the q8, q4, and IV induction groups appeared to be stable at the first and second follow-up compared to baseline. Notably, our study observed a high percentage of patients in clinical remission, ranging between 64% and 100% for CD and 70% and 100% for UC at various time points. A non-significant trend towards a lower proportion of patients in clinical remission was noticed in the q4 group. This population represents patients more refractory to therapy, which already needed to be optimised due to insufficient response. Another possible explanation might be the favourable safety profile of vedolizumab. Many elderly patients, patients with comorbidities or cancer, and patients wary of side effects tend to continue vedolizumab due to its selective mechanisms of action, despite only partial response to treatment. Similarly, in line with our findings, no statistically significant differences in clinical disease activity scores between baseline and follow-up were found in other studies [12,13,14,15]. For instance, in the Dutch study, the steroid-free clinical remission rates were 70%, 68%, and 39% for CD and 71%, 67%, and 44% for UC at baseline, week 12, and week 24, respectively [12]. Moreover, in the Swedish cohort, the clinical remission rates were 72%, 82%, and 73% in CD and 92%, 88%, and 92% in UC at switch, 3 months, and 6 months, respectively [13]. These percentages are comparable to our results.

No significant changes in CRP were observed at baseline and first and second follow-up across all patient groups. A similar trend was noted with FC, although our results were affected by missing data due to the retrospective nature of the study. Consistent with our findings, other real-world cohorts also reported no changes in biochemical parameters after switching to SC treatment [12,13,14,15]. However, a significant increase in FC after 12 weeks in the English cohort (from 31 µg/g to 47 µg/g) [14] and a significant decrease in FC after 6 months in the Swedish cohort (from 64 to 49 μg/g) were reported [15], both unlikely to be clinically relevant. Endoscopic remission in UC was achieved in the majority of patients: 89% in the q8, 67% in the q4, and 75% in the IV induction group. However, our analysis was again hampered by missing data due to the retrospective nature of the study and relatively short follow-up period. 

After transitioning to the SC formulation, median serum vedolizumab trough levels increased significantly from baseline in the q8 group (*p* < 0.001). However, no significant changes in vedolizumab serum concentrations between baseline and the first follow-up were found in the q4 group. These results could have been expected since patients in the q4 group received a double dose of IV vedolizumab compared to patients in the q8 group before transitioning to the same standard SC dose. Additionally, the q4 group represents the most refractory population of patients with high inflammatory burden who had inadequate or loss of response to standard vedolizumab dose every 8 weeks. Lower vedolizumab serum concentrations could reflect higher drug clearance due to active disease [6,24]. Similarly to our findings, the VISIBLE 1 study reported higher vedolizumab serum concentrations in the SC vedolizumab treatment group compared to the IV vedolizumab treatment group (infusions every 8 weeks) [8]. Notably, IV and SC formulations have different pharmacokinetic profiles. SC administration leads to incomplete bioavailability, gradual absorption, and lower peak concentrations, whereas IV infusion results in immediate systemic drug exposure and a high peak concentration [12,25]. However, the average drug exposure between 108 mg SC vedolizumab every 2 weeks and 300 mg IV vedolizumab every 8 weeks should be similar [12]. In our study, vedolizumab levels ranged between 22.7 and 28.6 µg/mL at week 14. These concentrations are comparable to those observed in the English (22.7 μg/mL at week 12) [14] and Swedish cohort (19.0 μg/mL at 6 months) [15] but lower compared to levels in the VISIBLE trials (34.6 μg/mL in the UC trial and 30.2 μg/mL in the CD trial) [8,9] and the Dutch cohort (31 μg/mL at week 12 and 37 μg/mL at week 24 in the Dutch cohort) [12].

While vedolizumab pharmacokinetics has been associated with clinical outcomes [4,5], the practical utility of measuring vedolizumab drug levels in clinical practice has been questioned. A vedolizumab serum concentration of 3 µg/mL induces a near-complete saturation of α4β7 on peripheral blood T cells [26]. Whether increasing vedolizumab concentrations (similar to concentrations with TNF antagonists) improves clinical outcomes remains unknown. After loss of response, approximately half of patients regain clinical response after dose escalation of IV vedolizumab by shortening the interval from 8 weeks to 4 weeks [6]. In real-world cohorts, more than half of patients on IV vedolizumab are optimised to infusions every 4 weeks [21]. However, Ungar at al. have argued against a pharmacokinetic basis for insufficient response to vedolizumab and questioned the need for dose escalation. In a retrospective study, they showed that lower vedolizumab levels before vedolizumab IV escalation were not predictive of success. On the contrary, higher pre-escalation vedolizumab levels were associated with better outcomes, possibly indicating lower inflammatory burden and higher probability of success [7,24]. Similarly, the endpoints of the ENTERPRET trial, comparing dose-optimisation strategy with vedolizumab to standard dosing in UC patients who had high drug clearance and exhibited primary nonresponse, were not met. The rates of endoscopic remission after 30 weeks were similar in the standard dosing arm (300 mg every 8 weeks) and optimised-dosing arm (300 mg or 600 mg every 4 weeks) [27]. The above findings are in line with our results. Patients optimised to 300 mg every 4 weeks due to insufficient response can be de-escalated to standard SC dosing (equivalent to 300 mg every 8 weeks) and maintain clinical and biochemical remission. 

Our real-world study, assessing the effectiveness of switching from IV to SC vedolizumab maintenance treatment in IBD patients, has several strengths. Notably, it represents the largest real-world cohort to date consisting of patients previously optimised due to loss of response or inadequate response by shortening IV dosing interval to every 4 weeks. Furthermore, our study has a longer follow-up period compared to other published real-world cohorts [12,14,15]. Due to the real-world nature of the study without exclusion criteria, our results reflect everyday clinical practice with a very heterogenous IBD population. It is the only published real-world cohort of transitioning to vedolizumab SC with endoscopic data. 

Our current study has several limitations. Firstly, its retrospective study design contributed to a substantial amount of missing data, posing challenges to the statistical analysis and comprehensiveness of our results. Secondly, follow-up visits were scheduled by the treating physician at varying time points after the switch. Consequently, clinical disease activity scores and laboratory testing were performed at different intervals following transition to SC, potentially influencing data consistency. Thirdly, our study did not have a comparator arm as it was an observational study. The results were compared to baseline parameters (IV treatment before switch). Fourthly, all enrolled patients had to be willing to switch to SC formulation, potentially introducing selection bias. More refractory patients may have been less prone to switch to SC vedolizumab. Fifthly, the scheduling of endoscopies by the treating physician, especially in patients with suspected active disease, might have influenced our endoscopic remission rates (which might explain low endoscopic remission rates in CD). Lastly, all patients were included from a tertiary centre, which may represent a more refractory IBD population, potentially limiting the generalizability of our findings. 

## 5. Conclusions

The results of our real-world study suggest that transitioning patients established on IV vedolizumab to SC appears effective and safe. Transitioning to SC vedolizumab maintenance treatment is feasible also in the refractory patient population, which had to be optimised by shortening the IV interval to every 4 weeks due to insufficient response to standard dosing. Notably, clinical and biochemical remission in patients transitioning from IV to SC vedolizumab appears to be maintained, regardless of the baseline vedolizumab dosing regimen (every 4 weeks versus every 8 weeks). Further prospective studies with longer follow-up periods are needed to confirm these findings.

## Figures and Tables

**Table 1:** Patient demographics and baseline characteristics.

	q8		q4		IV Induction	
	CD (*n* = 26)	UC (*n* = 39)	CD (*n* = 17)	UC (*n* = 24)	CD (*n* = 8)	UC (*n* = 21)
Age at first SC dose, median in years (range)	55 (39–63)	47 (41–65)	51 (40–61)	41 (30–66)	54 (30–70)	43 (24–53)
Male sex, *n* (%)	17 (63)	25 (64)	8 (47)	9 (38)	5 (63)	12 (57)
Disease duration: years (IQR)	9 (1.6–17.1)	10 (4–19)	16 (6–21)	6 (3–10)	12 (9–22)	10 (4–13)
CD location, *n* (%)						
L1 (ileal)	6 (23)		3 (18)		1 (13)	
L2 (colonic)	11 (44)		4 (24)		5 (63)	
L3 (ileocolonic)	9 (34)		10 (59)		2 (25)	
L4+ (isolated upper gastrointestinal disease)	3 (11)		1 (6)		1 (13)	
CD behaviour, *n* (%)						
B1 (non-stricturing/non-penetrating)	21 (81)		5 (30)		6 (75)	
B2 (stricturing)	3 (12)		6 (35)		1 (12.5)	
B3 (penetrating)	2 (7)		6 (35)		1 (12.5)	
Perianal disease	1 (4)		6 (35)		2 (25)	
UC extent, *n* (%)						
E1 (proctitis)		5 (13)		0		9 (43)
E2 (left-sided colitis)		14 (36)		8 (33)		4 (19)
E3 (pancolitis)		20 (51)		16 (67)		8 (38)
Smoking status, *n* (%)						
Current smoker	4 (15)	3 (8)	3 (18)	2 (8)	1 (13)	3 (14)
Previous smoker	5 (19)	8 (21)	4 (24)	3 (13)	0	1 (5)
Non-smoker	17 (66)	28 (71)	10 (59)	19 (79)	7 (87)	17 (81)
Previous therapy with biologic, *n* (%)	10 (39)	14 (36)	15 (88)	11 (46)	4 (50)	6 (29)
>1 prior biologic *n* (%)	5 (19)	6 (15)	8 (47)	1 (4)	3 (37)	0
Previous therapy with corticosteroids, *n* (%)	14 (54)	28 (72)	11 (65)	21 (88)	6 (75)	11 (52)
Duration of IV vedolizumab treatment, months (IQR)	28 (21–44)	18 (11–31)	25 (16–43)	22 (14–32)	/	/

Abbreviations: UC: ulcerative colitis; CD: Crohn’s disease; q8: intravenous vedolizumab every 8 weeks; q4: intravenous vedolizumab every 4 weeks; IV intravenous; IQR: interquartile range.

**Table 2:** Patients who discontinued vedolizumab SC due to active disease.

Group	Patient	Duration of SC Vedolizumab	Reason for Discontinuation
q4	CD, after right hemicolectomy	10 months	Endoscopically active disease (Rutgeerts i2); asymptomatic
	CD, after ileo-caecal resection	6 months	Endoscopically active disease (Rutgeerts i4); asymptomatic
	UC	6 months	Clinically and endoscopically (endoscopic Mayo score 2) active disease
IV induction group	CD, after proctocolectomy	13 months	Endoscopically active disease (ulcers in the stomach and small bowel); asymptomatic
	UC	11 months	Endoscopically active disease (endoscopic Mayo score 3); asymptomatic
	UC	11 months	Clinically active disease

Abbreviations: UC: ulcerative colitis; CD: Crohn’s disease; q4: intravenous vedolizumab every 4 weeks; SC: subcutaneous.

**Table 3:** Clinical and biochemical disease activity.

	q8		q4		IV Induction	
	CD (*n* = 26)	UC (*n* = 39)	CD (*n* = 17)	UC (*n* = 24)	CD (*n* = 8)	UC (*n* = 21)
Median HBI (IQR; *n*)						
At baseline	1 (0–2; 18)		2 (0–6; 11)		1 (0–3; 7)	
At 1st FU	1 (0–2; 20)		1 (0–3; 15)		0 (0–1; 7)	
At 2nd FU	1 (0–2; 11)		3 (0–6;7)		1 (/;2)	
HBI < 5 (%)						
At baseline	17/18 (94)		7/11 (64)		7/7 (100)	
At 1st FU	20/20(100)		13/15 (87)		7/7 (100)	
At 2nd FU	10/11 (91)		5/7 (72)		2/2 (100)	
Median pMayo (IQR; *n*)						
At baseline		0 (0–1; 27)		1 (0–2; 17)		1 (0–2; 18)
At 1st FU		0 (0–1; 28)		0 (0–1; 17)		0 (0–1; 18)
At 2nd FU		0 (0–0; 13)		1 (1–3; 10)		1 (0–1; 9)
pMayo <2 (%)						
At baseline		26/27 (96)		12/17 (71)		13/18 (72)
At 1st FU		25/28 (89)		15/17 (88)		16/18 (89)
At 2nd FU		12/13 (92)		7/10 (70)		9/9 (100)
CRP, mg/L, median (IQR; *n*)						
At baseline	3 (3–9; 22)	3 (3–7; 30)	3 (3–6; 15)	3(3–11;23)	5 (3–17;7)	3 (3–3; 20)
At 1st FU	3 (3–4; 17)	3 (3–5; 30)	3 (3–3; 15)	3 (3–5; 17)	8 (3–8; 8)	3 (3–3; 18)
At 2nd FU	3 (3–5; 14)	3 (3–6; 12)	4 (3–6; 8)	4 (3–11;10)	7 (/; 2)	3 (3–3; 11)
CRP < 5 mg/L (%)						
At baseline	13/22 (59)	21/30 (70)	9/15 (60)	13/23 (57)	3/7 (37)	16/20 (80)
At 1st FU	13/17 (76)	22/30 (73)	13/15 (87)	11/17 (65)	3/8 (38)	15/18 (87)
At 2nd FU	8/14 (57)	9/12 (75)	4/8 (50)	7/10 (70)	1/2 (50)	8/11 (73)
FC, mg/kg, median (IQR; *n*)						
At baseline	29 (16–61; 20)	16 (16–50; 24)	131 (16–252; 7)	174 (35–419; 14)	16 (16–324; 5)	156 (34–212; 15)
At 1st FU	39 (16–119; 9)	16 (16–16; 15)	48 (18–337; 8)	165 (53–330; 6)	125 (16–147; 3)	91 (16–221; 12)
At 2nd FU	49 (48–147; 7)	40 (27–135; 10)	274 (/; 2)	500 (/; 4)	/	27 (27–53; 6)
FC < 100 mg/kg (%)						
At baseline	16/22 (72)	20/24 (87)	3/17 (18)	5/14 (36)	3/5 (60)	5/15 (33)
At 1st FU	6/9 (67)	13/15 (87) 7/10 (70)	5/8 (63)	2/7 (28)	1/3 (33)	6/12 (50)
At 2nd FU	3/7 (50)		1/2 (50)	0/4 (0)	/	5/6 (83)

Abbreviations: UC: ulcerative colitis; CD: Crohn’s disease; q8: intravenous vedolizumab every 8 weeks; q4: intravenous vedolizumab every 4 weeks; IV Induction: intravenous vedolizumab as an induction—2 or 3 infusions; HBI: Harvey Bradshaw index; pMayo: partial Mayo score; FU: follow up; CRP: C-reactive protein; FC faecal calprotectin; IQR: interquartile range; *n*: number of patients.

**Table 4:** Endoscopic disease activity.

	q8		q4		IV Induction	
	CD (*n* = 26)	UC (*n* = 39)	CD (*n* = 17)	UC (*n* = 24)	CD (*n* = 8)	UC (*n* = 21)
Median time to endoscopy after switch to SC in weeks (IQR)	24.5 (17–42)		29 (21–42)		21 (16–29)	
Endoscopic remission (percentage)	1/1 (100)	8/9 (89)	0/5 (0)	4/6 (67)	0/2 (0)	9/12 (75)

Endoscopic remission was defined as Mayo endoscopic score < 2 in UC and the absence of ulcers in CD. Abbreviations: UC: ulcerative colitis; CD: Crohn’s disease; q8: intravenous vedolizumab every 8 weeks; q4: intravenous vedolizumab every 4 weeks; IV induction: intravenous vedolizumab as an induction—2 or 3 infusions; IQR: interquartile range.

**Table 5:** Vedolizumab serum concentration at baseline and 1st and 2nd follow up.

	q8		q4		IV Induction	
	CD (*n* = 26)	UC (*n* = 39)	CD (*n* = 17)	UC (*n* = 24)	CD (*n* = 8)	UC (*n* = 21)
Vedolizumab serum concentration µg/mL (IQR. *n*)						
At baseline	10.9 (7.2–16.6; 46)		28.5 (17.3–42.1; 32)		26.0 (17–37; 23)	
At first follow-up	28.6 * (20.8–34.8; 7)		22.7 ** (17.9–29.9; 9)		25.3 *** (24.0–34.3; 11)	
At second follow-up	29.7 (22.4–36.4; 6)		19.0 (15.4–28.0; 6)		27.0 (18.9–32.3; 6)	

* *p* < 0.001, ** *p* = 0.575, *** *p* > 0.05. Abbreviations: UC: ulcerative colitis; CD: Crohn’s disease; q8: intravenous vedolizumab every 8 weeks; q4: intravenous vedolizumab every 4 weeks; IV induction: intravenous vedolizumab as an induction—2 or 3 infusions; IQR: interquartile range; *n*: number of patients.

## Data Availability

Data and materials available on request from the corresponding authors.
